# ProDTI: prior-guided positive-unlabeled learning for robust drug–target interaction prediction

**DOI:** 10.1093/bioinformatics/btag712

**Published:** 2026-10-07

**Authors:** Yu Wang, Haoxiang Zhang, Ziye Zhu, Qiang Wei, Yun Li

**Affiliations:** School of Artificial Intelligence, China Pharmaceutical University, Nanjing, 211198, China; School of Computer Science, Nanjing University of Posts and Telecommunications, Nanjing, 210023, China; School of Computer Science, Nanjing University of Posts and Telecommunications, Nanjing, 210023, China; Institute of Innovative Drug, China Pharmaceutical University, Nanjing, 211198, China; School of Pharmacy, China Pharmaceutical University, Nanjing, 211198, China; School of Computer Science, Nanjing University of Posts and Telecommunications, Nanjing, 210023, China

## Abstract

**Motivation:**

Accurate Drug–Target Interaction (DTI) prediction is critical for drug discovery. While deep learning advances this field, the fundamental lack of verified negative interactions inevitably causes biased risk estimation. Although formulating DTI prediction as a Positive-Unlabeled (PU) learning problem is theoretically sound, directly applying PU learning to deep models suffers from early-stage optimization instability and decision boundary ambiguity.

**Results:**

We propose ProDTI, a prior-guided PU learning framework integrating domain knowledge into the PU objective. We construct a hybrid prior combining explicit biochemical similarities with implicit semantic representations from large-scale pre-trained models. This prior drives two synergistic mechanisms: soft-label distillation to stabilize the optimization trajectory, and hard-anchor regularization to explicitly penalize reliable negatives and sharpen decision boundaries. Evaluated on five benchmarks, ProDTI consistently outperforms state-of-the-art baselines, exhibiting superior robustness in challenging cold-start scenarios and providing a reliable tool for computational drug discovery.

**Availability and Implementation:**

Source code and data are available at https://github.com/WangYu-AI4SCI/ProDTI.

## 1 Introduction

Drug–Target Interaction (DTI) prediction, which aims to identify binding affinities between candidate drugs and protein targets, is a fundamental task in pharmaceutical research (Yamanishi *et al.* 2008, Wang *et al.* 2024). Given the vast chemical and biological space, computational DTI prediction is essential to prioritize high-potential candidates, thereby reducing the cost and duration of wet-lab experimental validation (Chen *et al.* 2018, Vamathevan *et al.* 2019, Li *et al.* 2024). Recent studies have documented substantial progress in deep DTI models (Nunes *et al.* 2025), many of which employ a dual-tower architecture to encode drugs and targets separately (Meng *et al.* 2024, Hua *et al.* 2025).

Despite these architectural advancements, a key limitation remains in the prevailing training paradigm: an over-reliance on supervised learning, which assumes access to labeled positive and negative samples (i.e. verified interacting and non-interacting drug-target pairs) with a relatively balanced distribution. However, in practice, experimentally verified interactions are scarce, and true negatives are difficult to obtain (Fig. 1a). As a result, existing methods resort to random negative sampling, which inevitably introduces false negatives into training data and leads to biased empirical risk estimation (Fig. 1b). This bias is particularly pronounced in cold-start scenarios involving novel drugs or orphan targets.

**Figure 1:**
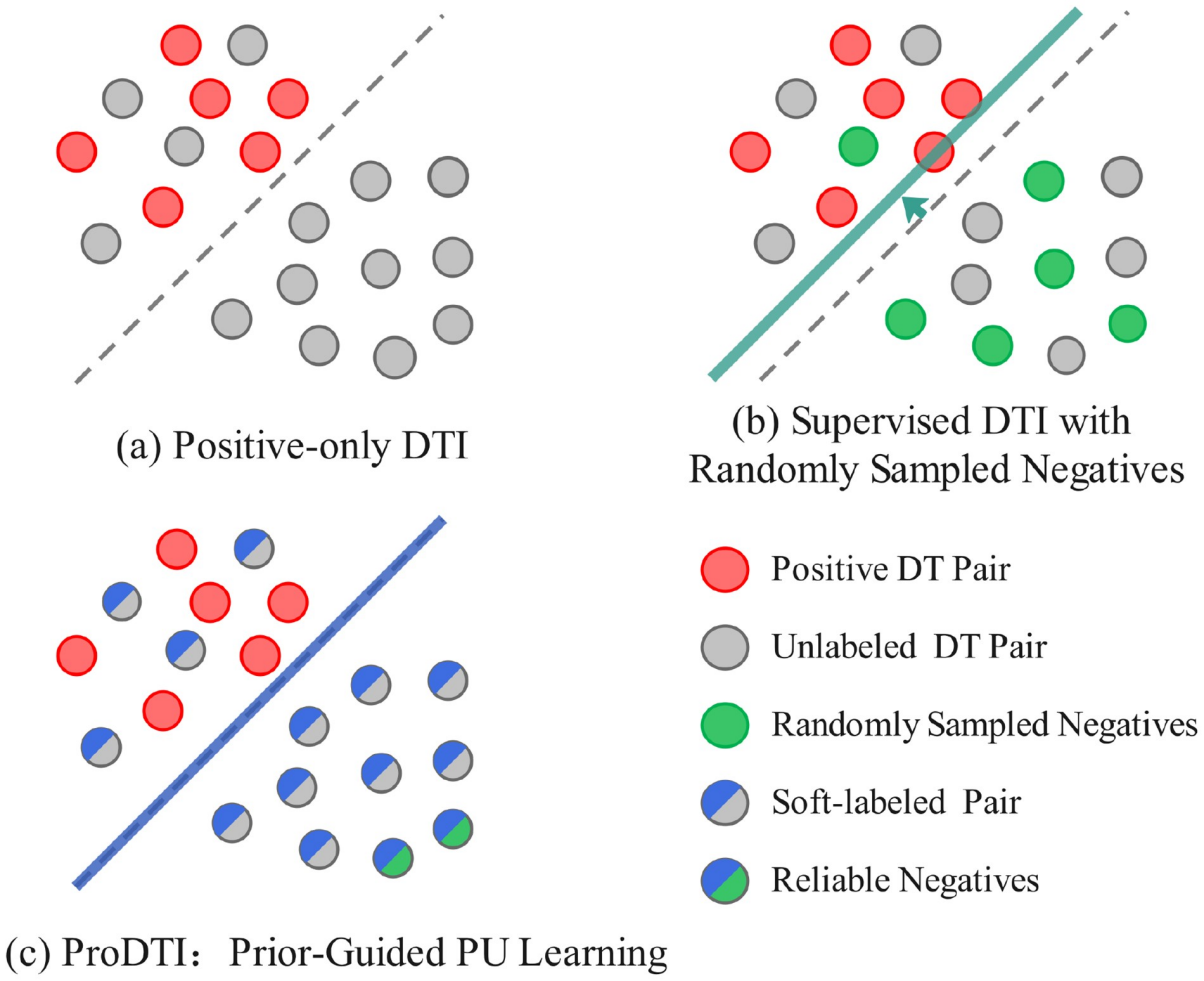
Comparison of different learning paradigms for DTI prediction. (a) *Positive-only DTI*: In real-world settings, only experimentally verified drug–target interactions are labeled as positives (red), while all other pairs remain unlabeled (grey). (b) *Supervised DTI with Randomly Sampled Negatives*: Unlabeled pairs are randomly treated as negative samples (green), which induces biased decision boundaries. (c) *ProDTI: Prior-Guided PU DTI: ProDTI* leverages domain priors to assign soft labels for knowledge distillation and identifies reliable negatives to refine the decision boundary.

Consequently, DTI prediction is more naturally formulated as a Positive-Unlabeled (PU) learning problem (Lan *et al.* 2016). Various PU learning algorithms have been applied to biological tasks (Zhang *et al.* 2020, Yan *et al.* 2021, Li *et al.* 2022). While such early PU-based approaches (Lan *et al.* 2016, Keum and Nam 2017, Peng *et al.* 2017) attempt to identify reliable negatives via heuristic strategies, their rigid criteria frequently fail to capture complex biochemical semantics and functional correlations (Song *et al.* 2025). In contrast, non-negative PU (nnPU) (Kiryo *et al.* 2017) offers a theoretically unbiased alternative by optimizing risk over the entire unlabeled distribution. Nevertheless, directly applying nnPU to deep DTI models aggravates its inherent optimization risks. Specifically, the strict non-negative constraint tends to induce instability during the early training phase (Kiryo *et al.* 2017), while the lack of pair-level supervision results in ambiguous decision boundaries, particularly problematic in the vast and sparse DTI search space (Chen *et al.* 2020).

To address these challenges, we propose a novel deep PU learning framework named ProDTI (Prior-guided PU learning for robust DTI prediction). The backbone of our framework consists of a dual-tower encoder optimized by an nnPU unbiased risk estimator. To alleviate the optimization instability and decision boundary ambiguity, we introduce two prior-driven modules: Hybrid Prior Construction and Prior Knowledge Injection. The Hybrid Prior Construction module aims to assign domain prior scores to all unlabeled drug-target pairs. Specifically, grounded in the “guilt-by-association” principle, we evaluate each unlabeled pair by aggregating association signals from its verified positive neighbors on both the drug and target sides. To quantify these priors, we integrate two complementary perspectives: explicit biomedical similarity [(e.g. Tanimoto coefficient (Rogers and Tanimoto 1960)] and implicit semantic similarity derived from large-scale pre-trained models [e.g. ESM-2 (Lin *et al.* 2023)]. These priors are effectively leveraged via the Prior Knowledge Injection module through two synergistic mechanisms: (i) soft-label distillation and (ii) hard-anchor constraints. Soft-label distillation transfers domain knowledge by treating prior scores as continuous “soft labels”, providing dense supervisory signals that guide the model to approximate the prior distribution. This mechanism effectively stabilizes the optimization trajectory against gradient vanishing, particularly during the early training phase. Concurrently, hard-anchor constraints identify reliable negatives from unlabeled pairs with minimal prior similarity. By imposing strict penalties on these “hard anchors”, we explicitly enforce discriminative decision boundaries to distinguish true interactions from vast non-interacting space. Extensive experiments on five benchmark datasets (DrugBank, BioSNAP, GPCRs, Ion Channel, and Davis) demonstrate that ProDTI significantly outperforms state-of-the-art baselines. Notably, it exhibits substantial gains in AUPR and F1-score.

- To the best of our knowledge, we provide the first systematic explorations of deep PU learning for DTI prediction, transitioning the task from biased random negative sampling to prior-guided risk minimization.
- We propose ProDTI, which incorporates hybrid priors into the nnPU objective through soft-label distillation and hard-anchor constraints.
- Extensive evaluations demonstrate the superiority of ProDTI, particularly in AUPR and F1-score, which reduces the risk of failed wet-lab validations, even in challenging cold-start scenarios.

## 2 Problem statement

We define the drug set as $D=\left\{d_{1},d_{2},\ldots ,d_{M}\right\}$ and the target set as $T=\left\{t_{1},t_{2},\ldots ,t_{N}\right\}$, where *M* and *N* are their sizes. Interactions of all drug-target pairs are summarized in a partially observed matrix $Y\in {\left\{1,\text{null}\right\}}^{M\times N}$, where $y_{i,j}=1$ indicates an interaction exists in $\left(d_{i},t_{j}\right)$, and $y_{i,j}=\text{null}$ denotes an unverified status. We denote all verified interacting pairs as the positive set P and unverified pairs as the unlabeled set U, where |P|≪|U|. The verified interacting targets of $d_{i}$ and drugs of $t_{j}$ are denoted as $T_{d_{i}}^{p}$ and $D_{t_{j}}^{p}$, respectively.

**Problem 1**. DTI Prediction under PU Setting

**Given:** (i) drug set D with M drugs; (ii) target set T with N targets; (iii) a partially observed matrix $Y\in {\left\{1,\text{null}\right\}}^{M\times N}$; (iv) A query pair (d,t) involving potentially novel drugs or targets.

**Goal**: Learn a scoring function f:D×T→[0,1] to estimate interaction probability and recover latent positives.

## 3 System and methods

Figure 2 illustrates the overall architecture of ProDTI. The framework is built upon a dual-tower PU backbone, which establishes the fundamental dual-tower DTI architecture and nnPU-based optimization under the positive-unlabeled setting. Built on this foundation, ProDTI further incorporates two prior-driven modules: Hybrid Prior Construction and Prior Knowledge Injection. Specifically, the former derives informative priors from explicit and implicit views, while the latter injects the constructed priors into PU optimization to alleviate optimization instability and decision boundary ambiguity caused by noisy unlabeled pairs.

**Figure 2:**
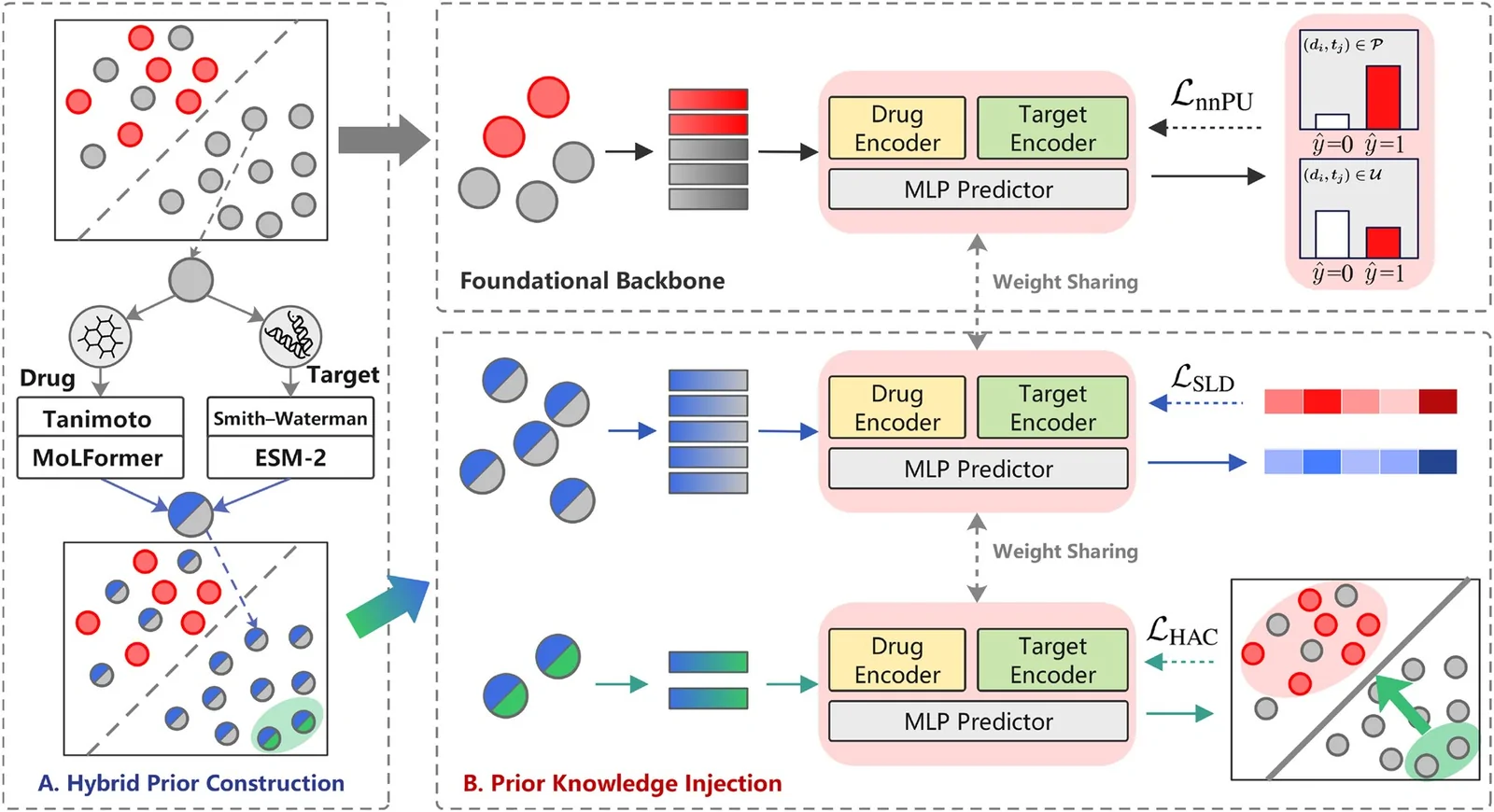
ProDTI consists of a dual-tower PU backbone and two prior-driven modules: (A) Hybrid Prior Construction and (B) Prior Knowledge Injection. The former utilizes hybrid domain knowledge to assign prior scores to all unlabeled pairs. These priors are subsequently leveraged via the Prior Knowledge Injection module, including soft-label distillation ($L_{\text{SLD}}$) to stabilize training and hard-anchor constraints ($L_{\text{HAC}}$) to sharpen decision boundaries.

### 3.1 Dual-tower PU backbone

The dual-tower PU backbone consists of a dual-tower encoder and an interaction predictor, and is trained under the nnPU learning paradigm. The dual encoder independently learns representations for drugs and targets via separate neural networks. Formally, given a drug $d_{i}$ and a target $t_{j}$, their raw features are mapped into latent representations in separate embedding spaces. Specifically, we employ two CNN-based encoders to extract structural features from drug SMILES sequences and protein amino acid sequences, respectively. The interaction probability ${\hat{y}}_{i,j}\in [0,1]$ is then estimated by an interaction predictor, which consists of a shared-weight multi-head cross-attention module followed by a prediction layer. To handle the positive-unlabeled learning setting, we adopt the nnPU objective to perform unbiased risk estimation over the unlabeled set U:


(1) $$\begin{array}{c} L_{\text{nnPU}}=\pi_{p}{\hat{L}}_{p}^{+}+\text{max}\left(0,{\hat{L}}_{u}^{-}-\pi_{p}{\hat{L}}_{p}^{-}\right),\\ \text{where}\;{\hat{L}}_{p}^{+}=\frac{1}{\left|P\right|}\sum_{(d_{i},t_{j})\in P}\ell ({\hat{y}}_{i,j},+1),\\ {\hat{L}}_{u}^{-}=\frac{1}{\left|U\right|}\sum_{(d_{i},t_{j})\in U}\ell ({\hat{y}}_{i,j},0),\\ {\hat{L}}_{p}^{-}=\frac{1}{\left|P\right|}\sum_{(d_{i},t_{j})\in P}\ell ({\hat{y}}_{i,j},0), \end{array}$$

where $\pi_{p}$ is positive class prior; ℓ is binary cross-entropy loss function.

### 3.2 Hybrid prior construction

To derive informative priors, we construct a hybrid prior scoring mechanism that fuses explicit biochemical similarity measures with implicit semantic representations to assign prior scores (i.e. soft labels) to unlabeled drug-target pairs. Specifically, given an unlabeled pair $\left(d_{i},t_{j}\right)$, we estimate its interaction potential based on the “guilt-by-association” principle. For each view v∈{bio,sem}, we compute drug-side (or target-side) similarities between $d_{i}$ (or $t_{j}$) and its respective known interaction partners $D_{t_{j}}^{p}$ (or $T_{d_{i}}^{p}$).

#### 3.2.1 Explicit biochemical view

The explicit view captures local structural and biochemical properties using classical bioinformatics descriptors. For drugs, we utilize Morgan fingerprints (Rogers and Hahn 2010) and compute similarity via Tanimoto coefficient (Rogers and Tanimoto 1960). Given drugs $d_{i}$ and $d_{k}$ with fingerprint vectors $\text{FP}(d_{i})$ and $\text{FP}(d_{k})$, the similarity between $d_{i}$ and $d_{k}$ is calculated as:


(2) $${\text{Sim}}_{D}^{\text{bio}}(d_{i},d_{k})=\frac{\left|\text{FP}(d_{i})\cap \text{FP}(d_{k})\right|}{\left|\text{FP}(d_{i})|+|\text{FP}(d_{k})|-|\text{FP}(d_{i})\cap \text{FP}(d_{k})\right|}.$$

Similarly, for targets, we use the Smith-Waterman algorithm (Smith and Waterman 1981) with normalization:


(3) $${\text{Sim}}_{T}^{\text{bio}}(t_{j},t_{m})=\frac{\text{SW}(t_{j},t_{m})}{\sqrt{\text{SW}(t_{j},t_{j})\cdot \text{SW}(t_{m},t_{m})}}.$$

#### 3.2.2 Implicit semantic view

The implicit view leverages large-scale pre-trained models to capture global semantic relations, complementing the explicit view by identifying functionally similar pairs that may share low structural overlap. We use MolFormer (Ross *et al.* 2022) as the drug encoder $E_{\text{MF}}(\cdot )$ and ESM-2 (Lin *et al.* 2023) as the target encoder $E_{\text{ESM}}(\cdot )$ to extract high-dimensional embeddings. The implicit similarity ${\text{Sim}}_{D}^{\text{sem}}(d_{i},d_{k})$ and ${\text{Sim}}_{T}^{\text{sem}}(t_{j},t_{m})$ is then quantified via the cosine distance:


(4) $$\begin{array}{c} {\text{Sim}}_{D}^{\text{sem}}(d_{i},d_{k})=\text{cos}(E_{\text{MF}}(d_{i}),E_{\text{MF}}(d_{k})),\\ {\text{Sim}}_{T}^{\text{sem}}(t_{j},t_{m})=\text{cos}(E_{\text{ESM}}(t_{j}),E_{\text{ESM}}(t_{m})). \end{array}$$

For each view v∈{bio,sem}, we compute the prior score by aggregating neighborhood similarities:


(5) $${\text{score}}_{i,j}^{v}=\frac{1}{2}(\underset{d_{k}\in D_{t_{j}}^{p}}{\text{max}}\;{\text{Sim}}_{D}^{v}(d_{i},d_{k})+\underset{t_{m}\in T_{d_{i}}^{p}}{\text{max}}\;{\text{Sim}}_{T}^{v}(t_{j},t_{m})),$$

where $D_{t_{j}}^{p}$ denotes the set of drugs having positive interactions with target $t_{j}$, and $T_{d_{i}}^{p}$ denotes the set of targets having positive interactions with drug $d_{i}$. The final prior score is obtained via weighted fusion:


(6) $${\text{score}}_{i,j}=\alpha \cdot {\text{score}}_{i,j}^{\text{bio}}+(1-\alpha )\cdot {\text{score}}_{i,j}^{\text{sem}},$$

where α is a hyperparameter balancing the contributions of explicit and implicit views. Importantly, the prior scores are derived exclusively from the training interactions and are used only during model training. Neither the neighborhood sets nor the prior scores involve any validation or test interactions, and no prior is computed at inference time.

### 3.3 Prior knowledge injection

This section mainly elaborates on the strategy for injecting the constructed hybrid prior into the foundational backbone. To leverage the domain knowledge derived from the explicit and implicit views, we introduce two synergistic injection mechanisms: soft-label distillation and hard-anchor constraints.

#### 3.3.1 Soft-label distillation

To regulate the model training with fine-grained structural information, we employ the knowledge distillation strategy. We treat prior scores as continuous soft targets to guide the manifold learning of the backbone. Specifically, during the training phase, we randomly sample a batch of samples $B^{u}$ from the unlabeled set U. The model is optimized to minimize the cross-entropy loss between the predicted probabilities and the pre-computed prior scores. The distillation loss $L_{\text{SLD}}$ is formulated as:


(7) $$\begin{array}{c} L_{\text{SLD}}=-\frac{1}{\left|B^{u}\right|}\sum_{(d_{i},t_{j})\in B^{u}}({\text{score}}_{i,j}\;\text{log}({\hat{y}}_{i,j})\\ +(1-{\text{score}}_{i,j})\;\text{log}(1-{\hat{y}}_{i,j})), \end{array}$$

where ${\hat{y}}_{i,j}$ denotes the model prediction for pair $\left(d_{i},t_{j}\right)$. This mechanism provides dense supervisory signals across the unlabeled space, which effectively stabilizes the optimization trajectory of the nnPU estimator, particularly in the early training stages.

#### 3.3.2 Hard-anchor constraints

While soft-label distillation provides global structural guidance, it may lack decisive boundaries for reliable negative regions. To enhance discriminability against non-interacting pairs, we identify reliable negatives (i.e. hard anchors) from the unlabeled set using the hybrid prior, defined as pairs with similarity scores below a strict threshold τ (e.g. τ<0.1). Then, we impose a constraint to force the model predictions for these anchors towards zero:


(8) $$L_{\text{HAC}}=-\frac{1}{\left|N_{\text{anchor}}\right|}\sum_{(d_{i},t_{j})\in N_{\text{anchor}}}\;\text{log}\;(1-{\hat{y}}_{i,j}),$$

where $N_{\text{anchor}}=\{(d_{i},t_{j})\in U|{\text{score}}_{i,j}<\tau \}$ represents the set of identified hard anchors. This constraint explicitly penalizes the model for assigning high interaction probabilities to structurally and semantically dissimilar pairs, thereby reducing the false-positive rate.

### 3.4 Training objective

To balance biological prior guidance with the discriminative capacity of the nnPU framework, we propose a dynamic hybrid loss,


(9) $$L(t)=\lambda (t)L_{\text{SLD}}+(1-\lambda (t))L_{\text{nnPU}}+\beta L_{\text{HAC}},$$

where $L_{\text{SLD}}$ in Eq.(7), $L_{\text{nnPU}}$ in Eq.(1), $L_{\text{HAC}}$ in Eq.(8), *t* denotes the training epoch, and β is a fixed coefficient. λ(t) is a time-dependent weighting parameter scheduled by cosine decay,


(10) $$\lambda (t)=\lambda_{\text{min}}+\frac{1}{2}(\lambda_{\text{max}}-\lambda_{\text{min}})\left(1+\text{cos}\;\left(\frac{t\pi }{T}\right)\right),$$

where $\lambda_{\text{max}}(=1$) and $\lambda_{\text{min}}(=0.5$) denote the initial and final weights, and *T* is the total number of training epochs. λ(t) gradually shifts the optimization from $L_{\text{SLD}}$ to $L_{\text{nnPU}}$.

## 4 Experimental results and analysis

### 4.1 Setup

*Datasets.* To evaluate our framework, we conduct extensive experiments on five widely recognized DTI benchmarks: DrugBank, BioSNAP, GPCRs, Ion Channel, and Davis. The dataset statistics are summarized in Table 1.

**Table 1:** Dataset statistics.

Dataset	Drugs	Targets	Total pairs	Pos. (Verified)	Neg. (Verified)	Unlabeled	
						Neg. (Sampled)	Unknown (Remaining)
DrugBank	6 647	4 294	2 85 42 218	17 511	0	17 511	2 85 07 196
BioSNAP	4 510	2 182	98 36 310	13 836	0	13 647	98 08 846
GPCRs	567	296	1 67 832	3 098	0	3 099	1 61 635
Ion Channel	210	204	42 840	1 475	0	1 475	39 890
Davis	68	379	25 772	7 320	18 452	0	0

Neg. (Sampled) denotes unverified negatives constructed via random sampling from the unlabeled pairs; the remaining unselected pairs are excluded from the experiments.

For the first four datasets, we follow standard evaluation protocols (Öztürk *et al.* 2018, Zhao *et al.* 2022) and use the commonly adopted balanced evaluation setting, where unlabeled drug-target pairs are randomly sampled as pseudo-negatives at a 1:1 ratio with verified positive pairs. Because these pseudo-negatives are not experimentally verified non-interactions, they may contain latent positives and introduce false-negative noise.

To enable a controlled PU-learning evaluation, we additionally included the fully annotated Davis dataset. It contains 68 drugs and 379 targets, resulting in 25 772 fully labeled drug-target pairs. We split the Davis dataset into training, validation, and test sets at the drug-target pair level using a stratified 8:1:1 ratio. Specifically, we modify only the training split by retaining a fraction c∈{0.1,0.7} of positives as labeled, while mixing the remaining positives with verified negatives into an unlabeled pool. The validation and test splits remain fully annotated. Statistics of the resulting PU variants of Davis are reported in Table 2. Additional details regarding these datasets are provided in the Supplementary Material.

**Table 2:** Statistics of the fully annotated Davis benchmark and its PU-learning configurations used in our experiments.

Setting	Configuration	Training Set (80%)			Validation Set (10%)	Test Set (10%)
		Pos. (Verified)	Neg. (Verified)	Unlabeled	Fully Annotated	Fully Annotated
Ground Truth*	Davis	5856	14 762	0	2577	2577
PU Variants	Davis (c=0.7)	4099	0	16 519	2577	2577
	Davis (c=0.1)	586	0	20 032	2577	2577

* *Ground Truth* represents the original fully-annotated dataset, serving solely as the base for PU simulation and not included in our experimental evaluations.

*Baselines.* We compare our proposed ProDTI with several state-of-the-art DTI methods: MolTrans (Huang *et al.* 2021), HyperAttentionDTI (Zhao *et al.* 2022), MGraphDTA (Yang *et al.* 2022), MCANet (Bian *et al.* 2023), DrugBAN (Bai *et al.* 2023), BINDTI (Peng *et al.* 2025), SaeGraphDTI (Zhang *et al.* 2025), and GNNBlockDTI (Deng *et al.* 2025). Additionally, we evaluated two deep PU-learning-based DTI variants, namely Dual-tower nnPU and Dual-tower RNPU. A detailed description of each baseline is provided in the supplementary material.

*Evaluation Metrics.* We evaluate model performance using five standard metrics: Area Under the Precision-Recall Curve (AUPR), Area Under the ROC Curve (AUC), Precision (P), Recall (R), and F1-score (F1). For DrugBank, BioSNAP, GPCRs, and Ion Channel, the benchmark negatives are randomly sampled from unlabeled pairs, resulting in balanced evaluation sets that may still include latent positives. Therefore, metrics computed under this sampled-negative setting may be affected by false-negative noise and should be interpreted as comparative results under the conventional benchmark protocol. Among the reported metrics, AUC provides a relatively stable ranking-based reference because it evaluates the overall ordering between verified positives and sampled negatives without relying on a single classification cutoff. We regard AUC as the primary metric and report AUPR, P, R, and F1 as complementary metrics. For Davis, the validation and test sets are fully annotated and remain class-imbalanced, providing a cleaner evaluation setting. Accordingly, we regard AUPR as the primary metric, as it better reflects positive-class retrieval quality under imbalance, while AUC and F1 are reported as complementary metrics.

*Implementation Details.* We implement our model using PyTorch and perform our experiments on an NVIDIA RTX 4090 GPU. We randomly split the data into training, validation, and test sets five times. Each split was used for one independent training and evaluation run, and the final results are reported as mean ± standard deviation. Hyperparameters were tuned by random search on the validation set, and the final results were reported on the test set. The hyperparameter settings are shown in the Supplementary Material.

### 4.2 Main results

Table 3 presents the quantitative comparison on the large-scale DrugBank and BioSNAP datasets. Overall, ProDTI achieves the best performance across most metrics. On DrugBank, ProDTI outperforms GNNBlockDTI by 0.9% in AUPR and achieves a 2.0% absolute gain in Precision. This improvement indicates that our strategy effectively mitigates false positives from unlabeled noise. On BioSNAP, ProDTI delivers the highest AUPR (0.936) and F1-score (0.861). While MolTrans shows a slightly higher Recall, its Precision drops severely to 0.762. In contrast, ProDTI balances competitive Recall (0.862) with high Precision (0.860), which is critical for reducing downstream wet-lab validation costs.

**Table 3:** A comparison with the state-of-the-art methods on DrugBank and BioSNAP.

Method	**AUPR (std)** ↑	**AUC (std)** ↑	**P (std)** ↑	**R (std)** ↑	**F1 (std)** ↑
DrugBank					
MolTrans	0.887 (0.002)	0.878 (0.004)	0.786 (0.014)	0.824 (0.002)	0.804 (0.007)
HyperAttentionDTI	0.862 (0.007)	0.865 (0.003)	0.787 (0.011)	0.813 (0.018)	0.799 (0.009)
MGraphDTA	0.877 (0.006)	0.875 (0.004)	0.827 (0.014)	0.764 (0.025)	0.794 (0.007)
MCANet	0.904 (0.005)	0.898 (0.002)	0.809 (0.015)	0.810 (0.012)	0.809 (0.006)
DrugBAN	0.888 (0.003)	0.883 (0.003)	0.796 (0.016)	0.818 (0.006)	0.812 (0.003)
BINDTI	0.880 (0.003)	0.880 (0.001)	0.785 (0.014)	0.824 (0.006)	0.813 (0.001)
GNNBlockDTI	0.892 (0.003)	0.900 (0.004)	0.823 (0.005)	**0.841 (0.016)**	0.832 (0.004)
Dual-tower nnPU	0.893 (0.007)	0.881 (0.005)	0.826 (0.013)	0.781 (0.015)	0.804 (0.006)
Dual-tower RNPU	0.885 (0.007)	0.869 (0.007)	0.820 (0.012)	0.780 (0.017)	0.798 (0.007)
ProDTI	**0.922 (0.003)**	**0.911 (0.002)**	**0.853 (0.006)**	0.833 (0.007)	**0.843 (0.004)**
BioSNAP					
MolTrans	0.893 (0.004)	0.885 (0.001)	0.762 (0.013)	**0.869 (0.008)**	0.817 (0.003)
HyperAttentionDTI	0.927 (0.008)	0.912 (0.006)	0.825 (0.018)	0.857 (0.014)	0.841 (0.008)
MGraphDTA	0.905 (0.003)	0.902 (0.003)	0.837 (0.030)	0.804 (0.038)	0.819 (0.007)
MCANet	0.929 (0.003)	0.915 (0.002)	0.850 (0.019)	0.836 (0.009)	0.842 (0.005)
DrugBAN	0.909 (0.005)	0.905 (0.004)	0.826 (0.015)	0.842 (0.010)	0.838 (0.003)
BINDTI	0.899 (0.003)	0.899 (0.002)	0.814 (0.018)	0.841 (0.009)	0.834 (0.003)
GNNBlockDTI	0.921 (0.001)	0.918 (0.002)	0.855 (0.010)	0.853 (0.011)	0.854 (0.002)
Dual-tower nnPU	0.910 (0.007)	0.903 (0.004)	0.853 (0.013)	0.808 (0.014)	0.830 (0.008)
Dual-tower RNPU	0.905 (0.006)	0.892 (0.010)	0.852 (0.015)	0.803 (0.020)	0.828 (0.010)
ProDTI	**0.936 (0.003)**	**0.927 (0.002)**	**0.860 (0.008)**	0.862 (0.007)	**0.861 (0.004)**

**Bold** and underlined values denote the best and second-best performance, respectively.

Table 4 summarizes the performance on two small domain-specific datasets, the GPCRs and Ion Channel, which are more challenging due to sample scarcity. On GPCRs, ProDTI surpasses SaeGraphDTI by 1.0% in AUPR and 6.3% in F1-score. On Ion Channel, ProDTI achieves a remarkable AUPR of 0.820, outperforming SaeGraphDTI by 3.2%. The significant lead in AUPR and AUC confirms the capacity of ProDTI in global candidate ranking.

**Table 4:** Performance comparison on GPCRs and Ion Channel.

	**GPCRs**			**Ion Channel**		
Method	AUPR (std)↑	AUC (std)↑	F1 (std)↑	AUPR (std)↑	AUC (std)↑	F1 (std)↑
MolTrans	0.602 (0.032)	0.639 (0.028)	0.694 (0.027)	0.642 (0.035)	0.717 (0.021)	0.737 (0.017)
HyperAttentionDTI	0.706 (0.015)	0.730 (0.029)	0.694 (0.027)	0.722 (0.038)	0.788 (0.022)	0.788 (0.012)
MCANet	0.616 (0.019)	0.668 (0.011)	0.657 (0.017)	0.712 (0.019)	0.770 (0.018)	0.754 (0.021)
SaeGraphDTI	0.708 (0.017)	0.743 (0.019)	0.696 (0.007)	0.788 (0.018)	0.814 (0.012)	**0.821 (0.011)**
Dual-tower nnPU	0.677 (0.019)	0.707 (0.012)	0.695 (0.013)	0.700 (0.019)	0.775 (0.015)	0.727 (0.026)
Dual-tower RNPU	0.670 (0.013)	0.709 (0.010)	0.691 (0.011)	0.702 (0.012)	0.760 (0.011)	0.760 (0.015)
ProDTI	**0.718 (0.017)**	**0.747 (0.011)**	**0.759 (0.016)**	**0.820 (0.016)**	**0.819 (0.018)**	0.807 (0.021)

**Bold** and underlined values denote the best and second-best performance, respectively.

Because DrugBank, BioSNAP, GPCRs, and Ion Channel use sampled negatives, we further evaluated ProDTI on the fully annotated Davis benchmark to provide a cleaner assessment. Table 5 reports the results on the simulated PU variants of Davis under two labeling rates (c∈{0.7,0.1}). From the experimental results, we can observe several key insights. First, ProDTI consistently outperforms all baselines across both settings, especially under the extreme label-scarce setting (c=0.1). In this case, where 90% of true positives are hidden in the unlabeled pool, standard supervised models (e.g. MolTrans, HyperAttentionDTI, BINDTI) obtain an F1-score of 0.000. This failure occurs because the standard CE loss forces models to treat the whole unlabeled set as negative, biasing predictions entirely toward the negative class. In contrast, ProDTI maintains a robust F1-score of 0.368 and the highest AUPR of 0.657. Second, both deep PU-learning-based DTI variants also fail with an F1-score of 0.000 under c=0.1. By integrating our full framework, ProDTI significantly restores prediction capabilities, validating the necessity of our proposed components. Third, as *c* decreases from 0.7 to 0.1, all methods exhibit performance degradation due to the reduction of observed supervisory signals. However, ProDTI degrades much more gracefully than the baselines, demonstrating its superior robustness against severe label sparsity.

**Table 5:** Performance comparison with state-of-the-art methods on Davis under different imbalance ratios.

	**Davis (** c=0.7 **)**			**Davis (** c=0.1 **)**		
Method	AUPR (std)↑	AUC (std)↑	F1 (std)↑	AUPR (std)↑	AUC (std)↑	F1 (std)↑
MolTrans	0.771 (0.013)	0.886 (0.009)	0.618 (0.021)	0.599 (0.012)	0.785 (0.017)	0.000
HyperAttentionDTI	0.752 (0.009)	0.870 (0.006)	0.620 (0.018)	0.583 (0.024)	0.756 (0.019)	0.000
MCANet	0.785 (0.011)	0.899 (0.004)	0.643 (0.010)	0.630 (0.013)	0.788 (0.010)	0.000
DrugBAN	0.788 (0.007)	0.900 (0.005)	0.698 (0.014)	0.635 (0.014)	0.800 (0.009)	0.129 (0.045)
BINDTI	0.776 (0.006)	0.892 (0.004)	0.668 (0.012)	0.600 (0.018)	0.775 (0.014)	0.000
GNNBlockDTI	0.779 (0.006)	0.888 (0.004)	0.712 (0.008)	0.616 (0.015)	0.796 (0.010)	0.172 (0.051)
Dual-tower nnPU	0.784 (0.010)	0.896 (0.005)	0.628 (0.024)	0.611 (0.016)	0.783 (0.015)	0.000
Dual-tower RNPU	0.763 (0.009)	0.878 (0.004)	0.698 (0.013)	0.618 (0.015)	0.786 (0.009)	0.000
ProDTI	**0.805 (0.011)**	**0.904 (0.005)**	**0.719 (0.006)**	**0.657 (0.015)**	**0.816 (0.005)**	**0.368 (0.034)**

**Bold** and underlined values denote the best and second-best performance, respectively.

### 4.3 Performance analysis in unseen settings

To evaluate the robustness of ProDTI in real-world prediction scenarios, we adopt two standard experimental settings for new drugs or targets on the BioSNAP datasets:

- New-Drug: We randomly hold out 20% of the drugs as the test set, ensuring all associated interactions are excluded from training. The remaining 80% of drugs and their interactions are further divided into training and validation sets.
- New-Target: Similarly, 20% of the targets and all associated interactions are reserved for testing, while the remainder are divided into training and validation sets.

Notably, the hybrid prior is constructed only from the training interactions within each split and is used exclusively during model training. No validation or test interactions are involved in this step. New drugs or targets do not require neighborhood information or prior scores during inference.

Figure 3 illustrates the comparative performance under the unseen settings on BioSNAP. As observed, ProDTI consistently outperforms all baselines across all metrics in both cold-start scenarios. Specifically, in the New-Drug setting, ProDTI achieves an AUC of 0.897 and an AUPR of 0.914, surpassing the strongest competitor, GNNBlockDTI. The New-Target setting presents a greater challenge, causing severe performance degradation in robust baselines like DrugBAN and BINDTI. Despite this, ProDTI maintains robustness with the highest AUC (0.857) and AUPR (0.862). Crucially, ProDTI achieves an optimal precision-recall balance, outperforming GNNBlockDTI by 5.2% in Precision at a minor cost of a 4.0% Recall reduction. This high-precision trade-off is highly desirable in virtual screening. It demonstrates that our hard-anchor constraints effectively filter out ambiguous predictions, ensuring higher reliability for top-ranked candidates. Practically, minimizing false positives directly reduces the financial and time costs of downstream wet-lab validations.

**Figure 3:**
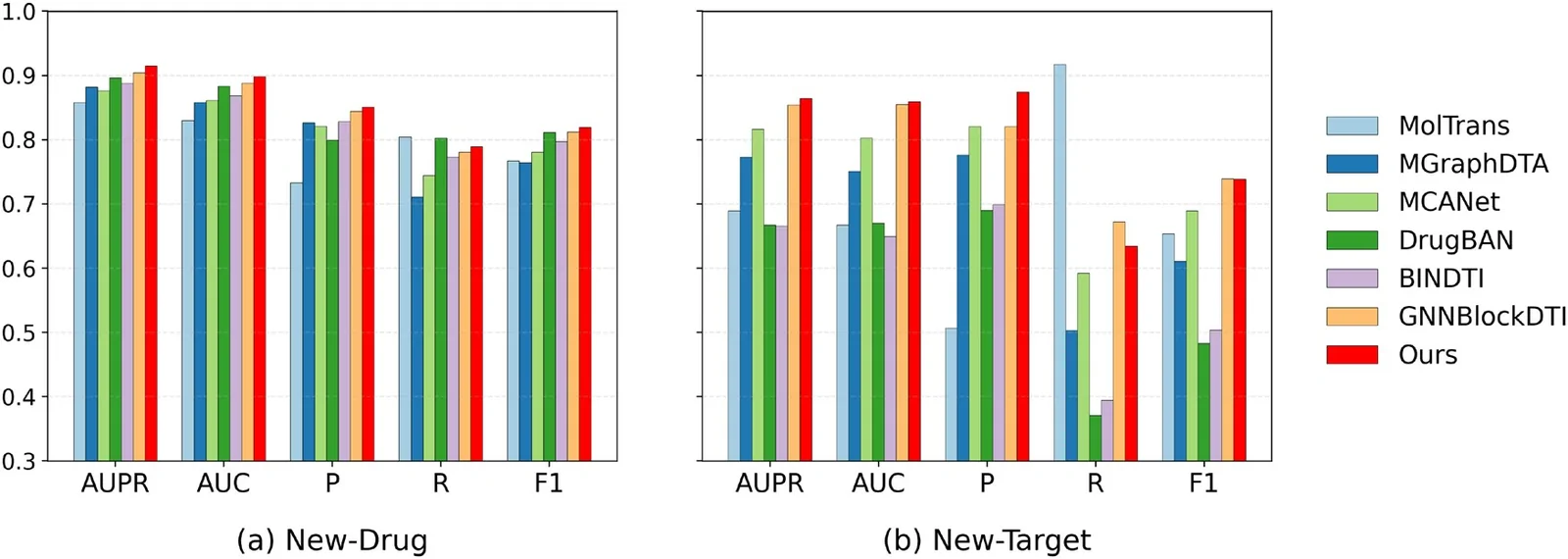
Performance evaluation under unseen settings on the BioSNAP dataset. (a) Ours vs. Fixed λ. (b) Ours vs. Dynamic λ.

### 4.4 Framework generality analysis

To further evaluate the generalization and adaptability of our prior-guided PU learning strategy, we integrate it as an alternative optimization objective into five distinct advanced DTI backbones while keeping their original feature extractors and predictors unchanged. Specifically, we replace the standard binary cross-entropy (BCE) objective with our PU risk estimator.

As illustrated in Table 6, our strategy consistently improves AUPR scores across all backbones, yielding an average absolute gain of 2.18%. This reveals that the false negative bias caused by random negative sampling is an architecture-agnostic limitation, regardless of whether a baseline relies on token-matching or graph message-passing. By refining risk estimation under the PU setting, our method enables different architectures to better distinguish true negatives from unlabeled pairs. More importantly, these substantial gains are achieved with zero parameter overhead. Because our enhancement is applied strictly at the optimization objective level, it introduces no additional neural layers or trainable weights. Therefore, our method delivers a pure and highly scalable algorithmic refinement that improves performance without compromising computational or memory efficiency.

**Table 6:** Plug-and-play enhancement results (AUPR) on DrugBank.

Baseline backbone	Original (BCE)	+ Our Strategy	Δ
MolTrans	0.887	0.905	+0.018
HyperAttentionDTI	0.862	0.895	+0.033
DrugBAN	0.888	0.903	+0.015
BINDTI	0.880	0.902	+0.022
GNNBlockDTI	0.892	0.913	+0.021
Average	–	–	+0.022

### 4.5 Component contribution analysis

Our proposed ProDTI integrates explicit biochemical indices and implicit semantic representations into backbone training through two synergistic mechanisms: soft-label distillation and hard-anchor constraints. To analyze the contribution of each mechanism, we construct three groups of ablation variants.

*(G1) Base Framework Evaluation:* (i) Dual-tower Sup Backbone: A standard supervised baseline that employs the same dual-tower encoder and is trained via BCE loss with randomly sampled negatives. (ii) Dual-tower PU Backbone: A vanilla non-negative PU baseline that employs the same dual-tower encoder and is optimized via the standard nnPU risk estimator without any prior guidance or constraints.

*(G2) Impact of Knowledge Sources:* (i) ProDTI-w/o-Bio: A variant that relies exclusively on implicit semantic representations by removing explicit biochemical indices. (ii) ProDTI-w/o-Sem: A variant that tests the sufficiency of biochemical indices.

*(G3) Efficacy of Injection Mechanisms:* (i) ProDTI-w/o-SLD: A variant that removes soft-label distillation while retaining only the hard constraints. (ii) ProDTI-w/o-HAC: A variant that operates without reliable hard constraints, relying solely on soft distillation.

The ablation study results on the DrugBank are summarized in Table 7. First, evaluating the Base Frameworks reveals that without domain-specific guidance, directly deploying unbiased risk estimators yields subpar results, tying with Dual-tower SUP Backbone at 0.892 in AUPR. This indicates that vanilla PU learning struggles to distinguish latent positives from the expansive unobserved space in complex DTI tasks, underscoring the necessity of incorporating structured prior knowledge. Second, regarding Knowledge Sources, the full ProDTI framework consistently outperforms both single-view variants (-w/o-Bio and -w/o-Sem). Notably, performance drops more severely when explicit biochemical indices are omitted (-w/o-Bio falling to 0.912). This emphasizes that explicit biochemical domains provide rigid, deterministic structural boundaries for optimization, while the implicit features derived from pre-trained biological models offer generalized functional contexts that fine-tune these boundaries. Finally, the Injection Mechanisms ablation clarifies how our optimization strategies stabilize training. On DrugBank, omitting the soft-label distillation (-w/o-SLD) causes a more pronounced performance drop (0.905 AUPR) than omitting the hard-anchor constraints (-w/o-HAC, 0.910 AUPR). This indicates that continuous, dense knowledge distillation is vital when dealing with sparse interactions, while both mechanisms together serve as vital tools for ensuring the model’s robustness.

**Table 7:** Ablation study results of ProDTI on DrugBank.

Group	Variant	AUPR	AUC
Base frameworks	Dual-tower Sup Backbone	0.892	**0.887**
	Dual-tower PU Backbone	0.893	**0.881**
Knowledge sources	ProDTI-w/o-Bio	0.912	0.905
	ProDTI-w/o-Sem	0.921	0.907
Injection mechanisms	ProDTI-w/o-SLD	0.905	0.894
	ProDTI-w/o-HAC	0.910	0.900
Ours	ProDTI (Full)	**0.922**	**0.911**

**Bold** values denote the best performance.

### 4.6 Parameter sensitivity analysis

The weighting factor λ(t) in ProDTI follows a cosine decay schedule (1→0.5). We compare it with:

- Fixed Weights: Fixed-High (λ=0.9), -Mid (λ=0.5), and -Low (λ=0.1), evaluating static trade-offs between prior regularization and empirical risk.
- Linear Decay: λ(t) decreases linearly from 1 to 0.5, assessing the effect of a uniform linear schedule.
- Cosine Decay: Cosine-Slow (1→0.8) and -Fast (1→0.1), examining sensitivity to the speed and range.

The performance comparison for λ(t) on DrugBank is illustrated in Fig. 4. We can observe that our proposed dynamic strategy consistently outperforms all fixed-weight baselines. This confirms that a static trade-off is insufficient to balance early-stage optimization stability and late-stage unbiased convergence. Furthermore, cosine decay consistently surpasses linear decay. Evaluations of different cosine ranges indicate that the model performance is sensitive to the decay speed and terminal weight. Overall, our final selection (1→0.5) achieves the optimal results.

**Figure 4:**
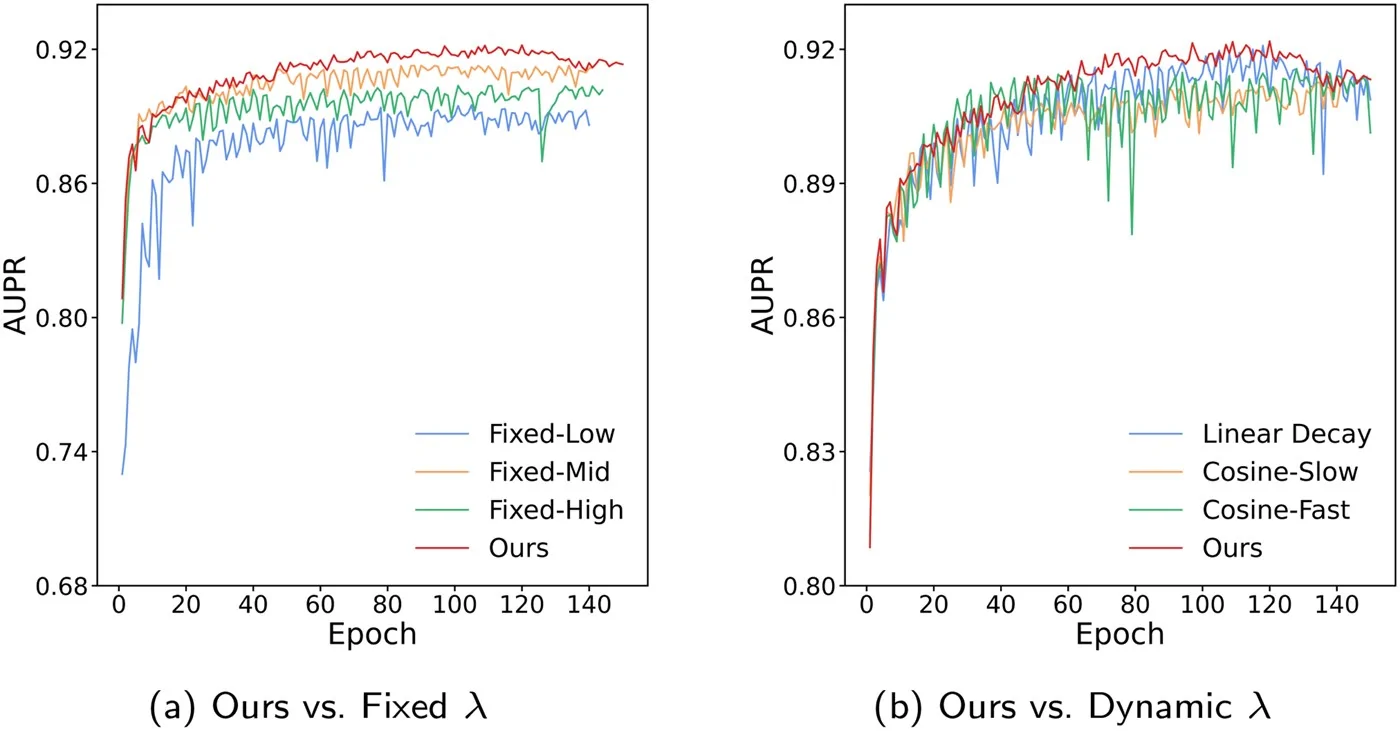
Sensitivity analysis of λ(t) on DrugBank dataset.

## 5 Conclusion

In this paper, we address DTI label sparsity by formulating it as a PU learning task. We identify that while theoretically unbiased, standard nnPU is compromised by early-stage instability and ambiguous decision boundaries in deep DTI models. To address these limitations, we propose a prior-guided framework ProDTI. ProDTI first constructs pair-level priors for unlabeled drug-target pairs by leveraging hybrid domain knowledge, and then injects these priors into risk minimization via soft-label distillation and hard-anchor constraints. Our method successfully bridges the gap between PU learning and deep DTI prediction. Extensive experiments demonstrate that ProDTI not only outperforms state-of-the-art baselines but also exhibits superior robustness in challenging cold-start scenarios. We believe this work provides a reliable paradigm for unbiased drug discovery and opens new avenues for applying PU learning to other biological tasks with scarce negative labels.

## Supplementary Material

btag712_Supplementary_Data

## Data Availability

The data underlying this article are available in its online supplementary material.
