# Supplementary material for: ProDTI: prior-guided positive-unlabeled learning for robust drug–target interaction prediction
Source: Bioinformatics. 2026 Oct 7;42(10):btag712. doi: 10.1093/bioinformatics/btag712 (PMC13676319; doi:10.1093/bioinformatics/btag712)
Supplement: Supplementary file 1 [file btag712_supplementary_data.pdf]

Supplementary Material

for "ProDTI: Prior-Guided Positive-Unlabeled Learning for Robust Drug-Target Interaction Prediction"

1. Datasets

We evaluate our model on five widely recognized DTI benchmarks.[1] To maintain consistency with prior work [1–3], the former four datasets are curated into a balanced setting by randomly sampling unknown pairs as negatives.

- DrugBank: a robust bioinformatics resource providing high-quality DTI sequences. Following [2], we filtered inorganic compounds and unrecognized SMILES, resulting in a dataset of 6 647 drugs and 4 294 proteins with 17 511 verified interactions.
- BioSNAP: derived from the Stanford Biomedical Network collection, containing 4 510 drugs and 2 181 target proteins [4]. In our processed version, it contains 13 836 positive and 13 647 negative drug–target interaction pairs.
- GPCRs: collected from the GLASS database, this dataset focuses on G-protein-coupled receptors [5], involving 567 drugs and 296 targets with 3 098 verified positive DTIs after preprocessing.
- Ion Channel: contains experimentally validated interactions between drugs and ion channel proteins [6]. It includes 210 drugs and 204 ion channel proteins with 1 475 known interactions in our processed version.

To ensure an unbiased evaluation, we incorporate the fully annotated Davis dataset. The completely labeled nature of this dataset enables us to precisely control the positive labeling rate, thereby adapting it to our PU-learning configurations. It is worth noting that the fully annotated validation set provides additional information for model selection, which may slightly influence the final test performance.

- Davis: a comprehensive dataset containing 68 kinase inhibitors and 379 kinases. It provides a total of 25 772 explicit samples, including 7 320 positive interactions, 18 452 negative interactions, and 0 unlabeled pairs [7].

2. Baselines

We evaluate the performance of ProDTI by comparing it against a diverse set of state-of-the-art DTI prediction methods, categorized as follows:

A. Sequence-based Models

- MolTrans [8]: employs Frequent Consecutive Subsequence (FCS) mining and a Transformer-based encoder to capture contextual interaction patterns from drug and protein sequences.

B. Attention-based Models

- HyperAttentionDTI [2]: utilizes CNN backbones integrated with a HyperAttention mechanism to model subsequence-level interactions.
- MCANet [3]: features a shared-weight multi-head cross-attention mechanism to capture bidirectional influences between drugs and targets.

C. Graph-CNN Hybrid Models

- MGraphDTA [9]: combines a multi-scale GNN (MGNN) for molecular graph representation with a multi-scale CNN (MCNN) for protein motif extraction.
- DrugBAN [10]: uses GCNs and CNNs to process drugs and proteins, respectively, employing a Deep Bilinear Attention Network (BAN) with domain adaptation to learn pairwise local interactions.

[1] The DrugBank and BioSNAP datasets used in this study were obtained from the GNNBlockDTI repository (https://github.com/Ptexys/GNNBlockDTI), while the GPCRs, Ion Channel, and Davis datasets were sourced from the MCANet repository (https://github.com/MrZQAQ/MCANet).

Table S1. Detailed Hyperparameter Settings

Hyperparameter	Value
Learning rate	1×10^{-4}
Batch size	64
Weight decay	1×10^{-4}
Drop out	0.2
Drug CNN kernel sizes	[4, 6, 8]
Protein CNN kernel sizes	[4, 8, 12]
Self convolution channels	40
Character embedding dimension	64
Maximum epochs	150
Early stopping	50
π_p	0.1
α	0.75
β	0.2
τ	0.1
$\lambda_{\max}$	1
$\lambda_{\min}$	0.5
Optimizer	AdamW

- BINDTI [11]: integrates GCN for drugs and an ACMix module (combining convolution and self-attention) for proteins via a bidirectional intent network.
- GNNBlockDTI [12]: a multi-scale approach using stacked GNNBlocks for drugs and residue-level graph networks for proteins to refine local binding fragments.
- SaeGraphDTI [13]: a graph-based model that combines sequence feature extractors with a similarity-supplemented relational network to predict edge existence via a graph decoder.

D. Deep PU-based Models

- Dual-tower nnPU: adopts the dual-tower architecture and is trained with the non-negative positive-unlabeled (nnPU) objective. It serves as the dual-tower PU backbone of ProDTI.
- Dual-tower RNPU: first identifies a set of reliable negatives (RN) from the unlabeled samples and subsequently treats them as negative samples to formulate the PU task as a conventional binary classification problem.

3. Experiment settings

The hyperparameter settings are shown in Table S1.

4. Evaluation Metrics

To evaluate the predictive performance of ProDTI, we employ several standard classification metrics. Given the Positive-Unlabeled (PU) nature of the DTI task, we prioritize metrics that effectively reflect ranking quality under potential label noise and class imbalance. Here, we use TP, FP, TN, and FN to represent the counts of true positives, false positives, true negatives, and false negatives, respectively.

Precision (P), Recall (R), and F1-score (F1). These metrics assess the exactness and completeness of the model's predictions. Precision quantifies the fidelity of predicted interactions, while Recall measures the model's ability to retrieve verified DTIs. The F1-score provides a balanced assessment by calculating the harmonic mean of Precision and Recall:

$$\mathrm{P} = \frac{TP}{TP + FP}, \tag{S1}$$

$$\mathrm{R} = \frac{TP}{TP + FN}, \tag{S2}$$

$$\mathrm{F1} = \frac{2 \cdot \mathrm{P} \cdot \mathrm{R}}{\mathrm{P} + \mathrm{R}}. \tag{S3}$$

AUC (Area Under the ROC Curve). AUC measures the model's overall discriminative capability by integrating the True Positive Rate (TPR) against the False Positive Rate (FPR) across all possible thresholds:

$$\mathrm{AUC} = \int_0^1 \mathrm{TPR}(FPR)\, d(FPR) \tag{S4}$$

where

$$\mathrm{TPR} = \frac{TP}{TP + FN}, \quad \mathrm{FPR} = \frac{FP}{FP + TN}. \tag{S5}$$

An AUC value closer to 1 indicates a superior ability to distinguish positive interactions from non-interactions [14].

AUPR (Area Under the Precision-Recall Curve). In the DTI context, the unlabeled set inherently contains hidden positive samples (false negatives). Consequently, AUPR serves as our primary metric, as it is more sensitive to the retrieval quality of the positive class and robust to severe class imbalance [15]. AUPR summarizes the trade-off between Precision and Recall:

$$\mathrm{AUPR} = \int_0^1 \mathrm{Precision}(R)\, dR. \tag{S6}$$

Unlike AUC, AUPR focuses on the density of true positives at the top of the ranked list, making it particularly suitable for PU learning where confirmed negative labels are unavailable.

5. Performance in Unseen Settings

Table S2 reports the experimental results under the challenging cold-start settings (New-Drug and New-Target) on the DrugBank dataset. As observed, ProDTI consistently outperforms all state-of-the-art baselines across all evaluation metrics in both scenarios. Notably, in the New-Drug setting, our method achieves a remarkable AUPR of 0.892, significantly surpassing the runner-up MCANet by a margin of 5.2%. A similar trend is observed in the New-Target setting, where ProDTI maintains a distinct lead with an AUPR of 0.863 and an AUC of 0.832. These substantial improvements confirm that our prior-guided PU learning strategy effectively enhances the model's generalization capability when predicting interactions for unseen molecular entities.

6. Analysis and Discussion

For the BioSNAP dataset, we conduct the same set of ablation experiments to verify the generality of our findings, including:

- Base Frameworks: Dual-tower Sup Backbone and Dual-tower PU Backbone to compare standard supervised training versus nnPU without prior guidance.
- Knowledge Sources: ProDTI-w/o-Bio and ProDTI-w/o-Sem to assess the contributions of explicit biochemical indices and implicit semantic representations.
- Injection Mechanisms: ProDTI-w/o-SLD and ProDTI-w/o-HAC to isolate the effects of soft-label distillation and hard-anchor constraints.

The results in Table S3 consistently align with those observed on the previous dataset, confirming that both the hybrid prior and the prior-guided optimization mechanisms contribute substantially to performance across different benchmarks.

Table S2. Performance evaluation under unseen settings on the DrugBank dataset.

Method	AUPR	AUC	P	R	F1
**New-Drug**					
GNN-CPI	0.690	0.660	0.650	0.520	0.578
GNN-PT	0.725	0.655	0.675	0.435	0.530
DE-DTI	0.730	0.710	0.705	0.535	0.608
DeepConv-DTI	0.725	0.700	0.685	0.530	0.598
TransformerCPI	0.745	0.705	0.730	0.540	0.621
GraphDTA	0.620	0.615	0.635	0.360	0.459
MCANet	0.840	0.831	0.782	0.664	0.718
**ProDTI**	0.892	0.871	0.860	0.727	0.788
**New-Target**					
GNN-CPI	0.725	0.715	0.670	0.615	0.641
GNN-PT	0.785	0.775	0.750	0.640	0.690
DE-DTI	0.795	0.785	0.740	0.645	0.689
DeepConv-DTI	0.795	0.790	0.735	0.700	0.717
TransformerCPI	0.800	0.795	0.730	0.745	0.737
GraphDTA	0.805	0.800	0.735	0.715	0.725
MCANet	0.824	0.796	0.808	0.615	0.699
**ProDTI**	0.863	0.832	0.836	0.683	0.752

Table S3. Ablation study results of ProDTI on BioSNAP dataset.

Group	Variant	AUPR	AUC
Base Frameworks	Dual-tower Sup Backbone	0.922	0.911
	Dual-tower PU Backbone	0.910	0.903
Knowledge Sources	ProDTI-w/o-Bio	0.928	0.919
	ProDTI-w/o-Sem	0.932	0.924
Injection Mechanisms	ProDTI-w/o-SLD	0.929	0.920
	ProDTI-w/o-HAC	0.926	0.915
**Ours**	**ProDTI (Full)**	**0.936**	**0.926**

Table S4. Sensitivity analysis on the weighting factor α on different datasets.

α	AUPR	AUC	P	R	F1
**DrugBank**					
0.30	0.913	0.905	0.850	0.808	0.828
0.50	0.917	0.908	0.840	0.823	0.831
0.70	0.916	0.908	0.845	0.833	0.838
0.75	0.922	0.911	0.853	0.833	0.843
0.80	0.920	0.909	0.848	0.830	0.837
**BioSNAP**					
0.30	0.927	0.917	0.845	0.856	0.851
0.50	0.927	0.918	0.842	0.858	0.851
0.70	0.932	0.923	0.848	0.866	0.855
0.75	0.936	0.927	0.860	0.862	0.861
0.80	0.930	0.923	0.853	0.865	0.858

7. Parameter Sensitivity Analysis

We evaluated the sensitivity of the weighting factor α in the hybrid prior construction to examine how the balance between the explicit biochemical view and the implicit semantic view affects performance. As summarized in Table S4, $\alpha = 0.75$ achieves the best overall performance on both DrugBank and BioSNAP. This result suggests that the explicit biochemical view provides the primary discriminative signal, while the implicit semantic view contributes complementary information. When α is increased to 0.80, the performance shows a slight decline, indicating that overemphasizing the explicit view may weaken the benefits of the implicit view. Overall, the results remain stable across nearby values of α, demonstrating the robustness of the proposed hybrid prior under different settings of this hyperparameter.

In addition, we further benchmarked the weighting factor $\lambda(t)$ on BioSNAP to validate the generality of our conclusions. As illustrated in Fig. S1, the evaluated schemes remain identical: Fixed Weights (High, Mid, Low), Linear Decay, and Cosine Decay (Slow, Fast). Consistent trends are observed, demonstrating that the dynamic cosine schedule reliably balances prior-guided regularization and nnPU optimization across different datasets, leading to improved convergence and performance.

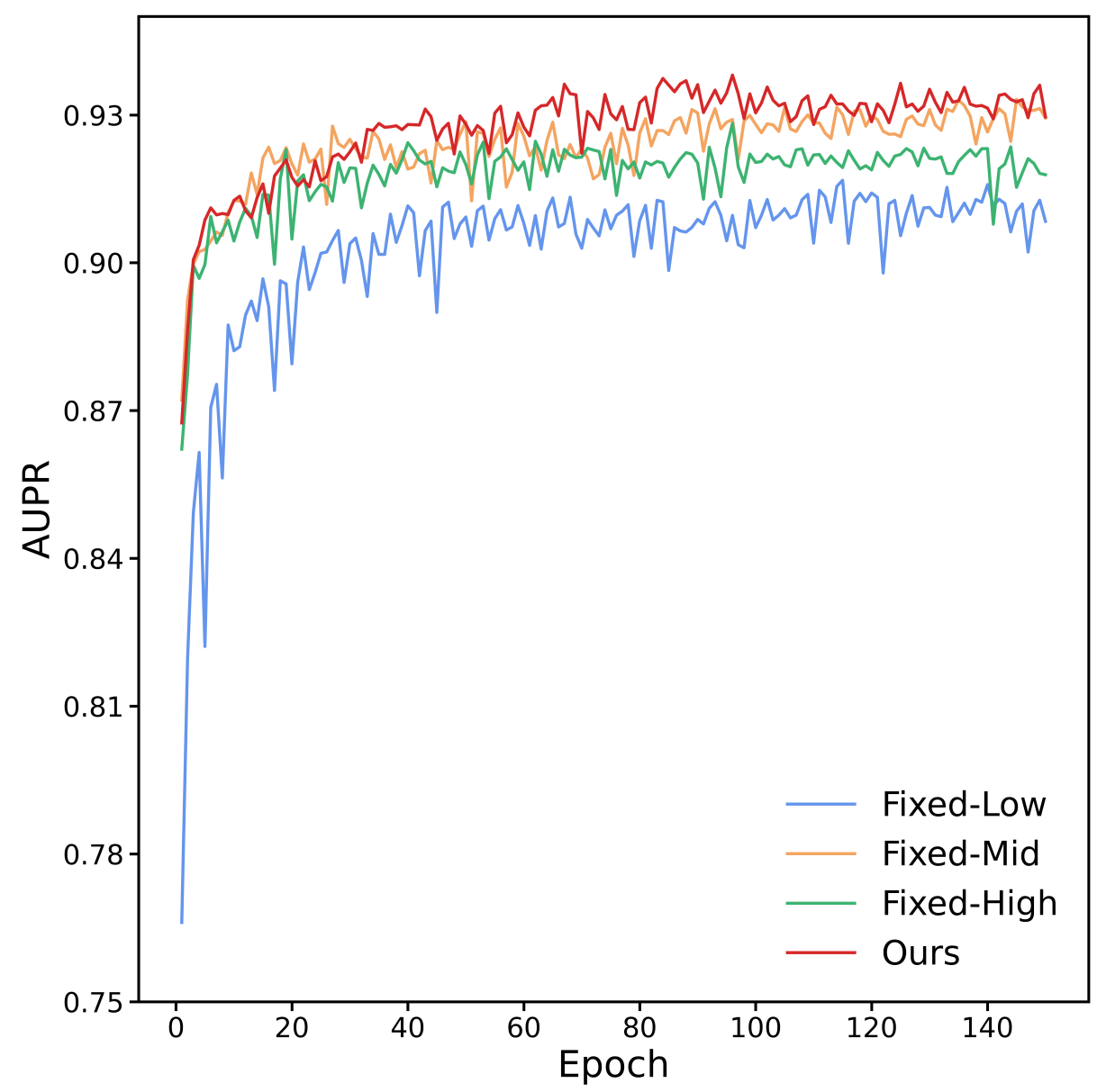

(a) Ours vs. Fixed λ

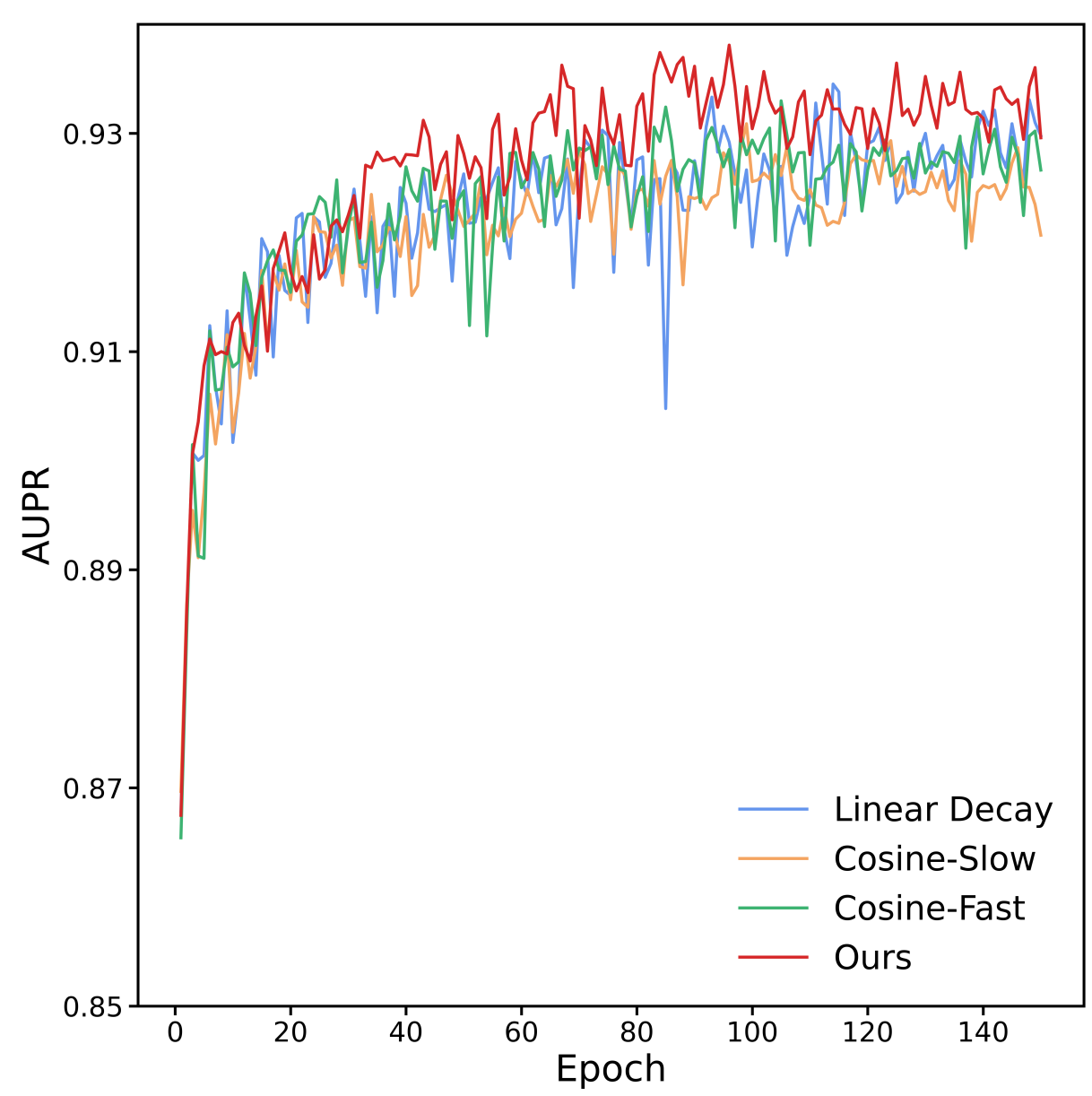

(b) Ours vs. Dynamic λ

Fig. S1. Sensitivity analysis on the weighting factor $\lambda(t)$ on BioSNAP dataset.

References

1. H. Öztürk, A. Özgür, and E. Ozkirimli, "Deepdta: deep drug–target binding affinity prediction," Bioinformatics **34**, i821–i829 (2018).
2. Q. Zhao, H. Zhao, K. Zheng, and J. Wang, "Hyperattentiondti: improving drug–protein interaction prediction by sequence-based deep learning with attention mechanism," Bioinformatics **38**, 655–662 (2022).
3. Z. Chen, L. Wang, H. Zhang, and M. Li, "Mcanet: Multi-channel attention network for drug–target interaction prediction," Briefings Bioinforma. **24**, bbad027 (2023).
4. M. Zitnik, R. Sosic, and J. Leskovec, "Biosnap datasets: biomedical network datasets for machine learning," Bioinformatics **34**, e1–e2 (2018).
5. W.-T. Chan, X. Li, R. Wang, *et al.*, "Glass: a comprehensive database of experimentally validated gpcr–ligand associations," Nucleic Acids Res. **43**, D1123–D1129 (2015).
6. Y. Yamanishi, M. Araki, A. Gutteridge, *et al.*, "Prediction of drug-target interaction networks from the integration of chemical and genomic spaces," Bioinformatics **24**, i232–i240 (2008).
7. M. I. Davis, J. P. Hunt, S. Herrgard, *et al.*, "Comprehensive analysis of kinase inhibitor selectivity," Nat. biotechnology **29**, 1046–1051 (2011).
8. K. Huang, C. Xiao, L. M. Glass, and J. Sun, "Moltrans: molecular interaction transformer for drug–target interaction prediction," Bioinformatics **37**, 830–836 (2021).
9. Z. Yang, W. Zhong, L. Zhao, and C. Y.-C. Chen, "Mgraphdta: deep multiscale graph neural network for explainable drug–target binding affinity prediction," Chem. science **13**, 816–833 (2022).
10. J. Zhu, Z. Shen, J. Lin, and S. Feng, "Drugban: A deep bilinear attention network for drug–target interaction prediction," Bioinformatics **38**, 4111–4119 (2022).
11. Y. Zhu, J. Li, X. Xu, and Y. Zhou, "Bindti: Bi-directional intention network for drug–target interaction prediction," Briefings Bioinforma. **24**, bbac532 (2023).
12. G. Deng, C. Shi, R. Ge, *et al.*, "Efficient substructure feature encoding based on graph neural network blocks for drug-target interaction prediction," Front. Pharmacol. **16**, 1553743 (2025).
13. X. Zhang, Y. Li, H. Wang, and Z. Chen, "Saegraphdti: Sequence attribute enhanced graph neural network for drug–target interaction prediction," Front. Pharmacol. **16**, 1553743 (2025).
14. T. Sakai, G. Niu, and M. Sugiyama, "Semi-supervised auc optimization based on positive-unlabeled learning," arXiv preprint arXiv:1705.01708 (2017).
15. J. Bekker and J. Davis, "Learning from positive and unlabeled data: a survey," arXiv preprint arXiv:1811.04820 (2018).
